# The burden among family caregivers of elderly cancer patients: prospective study in a Moroccan population

**DOI:** 10.1186/s13104-015-1307-5

**Published:** 2015-08-13

**Authors:** Sihame Lkhoyaali, Meryem Ait El Haj, Fadwa El Omrani, Mohammed Layachi, Nabil Ismaili, Hind Mrabti, Hassan Errihani

**Affiliations:** Medical Oncology Department, National Institute of Oncology, Rabat, Morocco; Department of Medical Oncology, University Hospital Mohammed VI, Marrakech, Morocco

**Keywords:** Caregivers, Burden, Elderly

## Abstract

**Background:**

In Morocco, families play a major role in caring for elderly cancer patients.

**Methods:**

We conducted a prospective descriptive study, in the National Institute of Oncology in Morocco. The study aimed to include family members who are caregivers for patients aged ≥70 years old.

**Findings:**

After obtaining IRB approval, a total of 150 caregivers responded to the questionnaire. Mean age was 44.7 years. The majority were females (59.3%), living in urban areas (66.7%), and educated (62.7%).Offspring (sons or daughters) represented 56.7, 54% lived with their relatives in the same house. Most of the participants were married and have familial responsibilities. In relatives, anxiety was found in 79.3%, it was related to fear of losing the patient in 57% and resulted in the use of anxiolytics in 10%. Guilt feeling towards patients regarding neglecting their early symptoms was reported in 38%. Depression and anxiety were more frequent among female relatives and among those of urban origin. Obsession of dying from cancer was present in about 30% and fear of contagion was more common among those from rural areas and illiterate. Economic resources were exceeded in 78.7 and 56% have used banking credits, and sale of properties. Work lay-off was recorded in 54%. Relatives participated in treatment making decisions in 86% of patients.

**Conclusion:**

Even there was a great impact on elderly cancerous patients relatives, the benefits of caregiving was observed in 80%. More studies have to be conducted, especially in developing countries where the lack of resources majors the impact on family caregivers.

**Electronic supplementary material:**

The online version of this article (doi:10.1186/s13104-015-1307-5) contains supplementary material, which is available to authorized users.

## Background

Supporting old cancer patients by their family caregivers is a difficult task, and the relatives are usually not prepared for such challenges. It could be a painful experience with a major emotional, physical and economical impact [1]. The motivation for informal caregiving is primarily voluntary and related to bonds of attachment between family members, but can also be affected by cultural norms regarding family obligations, or feelings of guilt towards relatives [2]. Elderly patients have special needs including assistance for medications, transportation for treatment sites and dealing with activities of daily living [3, 4].

In Morocco, family plays a major role in caring for elderly cancer patients. The majority of the elderly population lives with their relatives who provide the help and the care required. The impact on family caregivers of elderly cancer patients has not been evaluated before in a Moroccan population.

We conducted a explorative study to assess the social, psychological, behavioral and economic impact on patient’s family caregivers.

## Methods

This is a prospective descriptive study, conducted at the National Institute of Oncology in Rabat, Morocco from December 2011 to September 2012. The study was approved by the institutional review boards of the National Institute of Oncology, Cancer Centre in Rabat. We included relatives of patients aged ≥70 years old with histological confirmed cancer. A face-to-face interview conducted in the local language (Moroccan Arabic dialect) was completed by physicians participating in the study in collaboration with a psychologist. For all participants, demographics, disease characteristics, social, economical and psychological features were recorded. Psychological impact was assessed using DSM-IV: Diagnostic and Statistical Manual of Mental Disorders [5]. Informed oral consent was required.

The information was recorded in an Excel database and analyzed with the statistics software SPSS, version 12.0. Student test was used for quantitative parameters and Khi-2 for qualitative parameters. The difference between qualitative parameters was analyzed by non-parametric test. The risk factors were calculated by nonparametric test and logic regression.

## Results

### Caregivers characteristics

A total of 150 participants were included from all Moroccan regions. The mean age was 44 years, the majority was married, employed for full time, 53% of elderly patients lived with their families and 56% with the children (Additional file 1: Table S1).

Lung cancer, breast cancer and lymphoma are the most frequent cancer in patients.

### Psychological impact

Depression according to DSM-IV [5] was found in 34 participants (22.7%) and 94 (62.7%) reported at least one depressive signs. The depression was severe in four cases (2.7%).

Anxiety was often associated with depression, noted in 79.3% of cases and led to an anxiolytic taken in 15 caregivers (10%); it was linked to the fear of losing their close in 57.3% of cases.

Depression and anxiety were more common in urban origin and female relatives and, maybe because of women are more vulnerable and close to the relatives (Additional file 2: Table S2).

Guilt was objectified in 57 relatives (38%) due to a delay of consultation in 9.3% or neglect of the patient’s symptoms in 15.3%.

### Socio-behavioral impact of cancer in the family caregivers

Regarding the behavioral impact there were sleep disorders in 46% and loss appetite in 39.3% of cases. The obsession of having a cancer was present in 29.3% and the fear of contagion in 10.7%. The fear of contagion was more common in the rural illiterate population (Additional file 2: Table S2).

Fear of inheriting cancer was reported by daughters of women suffering from breast cancer. More than 32 of relatives reported that they are less socializing and neglect their families in 45% of cases.

### Economic impact of cancer in relatives

The economic families’ resources were exceeded in 78.7%. Thus, 56% reported having recourse to credits, 18.7% sale their goods, and 70.7% requested help from benefactors.

In addition, the support for these cancer patients caused the stoppage of work in 54% of cases; caregivers were obliged to quit their jobs in order to help the cancerous relatives (2 people were fired due to repeat absences from work).

### Participation in care and support benefits

Relatives play a vital role in the care of patients, they participated in making treatment decisions in 86% of cases, 75.3% wanted a maximalist treatment for their relatives and only 10.7% desired to limit a care. Despite the unfortunate consequences of cancer, 80% of families have reported a beneficial effect of support in particular overestimates the self.

## Discussion

Our study is the first study to be conducted in Morocco to evaluate the burden on elderly cancer patients’ caregivers.

Family Caregivers are involved in each step of the management including; patient follow-up, diagnosis announcement, treatment decision and side effects monitoring. They sometimes try to hide the diagnosis from the patient to overprotect him, which is frequent in our culture.

Studies indicates that while adult offsprings are major source of care for both older African Americans and Whites, African Americans are more likely to be cared for by a member of their extended family than Whites [6].

The support usually finished by the loss of a dear with all the ensuing stress and grief [3, 4, 7–9]. Cancer is a family’s illness because a diagnosis of cancer deeply affects the relationship and roles among family members. Anxiety and depression are the two of the most commonly reported problems for caregivers with estimates for depression at 39% and anxiety at 40% in several studies [10–14]. This impact differs depending on the stage of cancer [15, 16] and the type of support issued [17–19].

It is well established that female partners suffer more than men [20] and they have more depression [21] as it is demonstrated in our study. The spouses are more affected with more fatigue and insomnia because of their advanced age, their reduced physical ability and their strong desire to reduce the suffering of their partner [21–23].

Family caregivers report a negative social impact, particularly the disruption of their routines and alteration of their social relationships thinking that they have become less social [24, 25]. In our study this belief is present in 49 family (32.7%) and 69 reported negligence of the spouse and children and responsibilities towards the small family. In our country there is a lack of personnel medical care for elderly patient, the only source of care and nursing is the family. It is a shame and ungrateful in our culture to put a parent in the nursing home.

Other aspects of social and behavioral effects were noted in our population including the obsession with cancer (29.3%), fear of contagion (10.7%) and inherited cancer (20%) and anxiety of losing a close relative (57.3%), this is due to illiteracy and lack of medical information.

Some studies suggest that stress has a negative effect on the immunity, the blood pressure and the lipid metabolism [26–31]; this stress is considered by other authors as a risk factor for mortality among wives of elderly patients [32]. In a study in elderly patients with lung cancer reported that their wives had a physical health more impaired than in the control group [20]. In our study no physical impact of cancer has been identified in the families.

Caregiving may lead to hidden costs of care (negative effects experienced by the family members). It is estimated that 10 h per week are required to the support of a cancer patient over the age of 70, which corresponds to $ 1,200 per patient per year and more than a billion dollars in the United States [33]. In addition, we must consider the transportation and meals expenses, not reimbursed by social security [34–36]. In our context this economic cost is much more remarkable since 87.3% of our patients have no health coverage and 62.7% of the families asked have no monthly income or have a monthly income of less than $ 200.

The support of a patient with cancer has many negative effects; however it was beneficial in some cases. Caregiving has led to personal satisfaction, personality enhancement, having meaning in life and a greater appreciation of the family observed in 80% [20, 37, 38].

Our study supports the results of published studies that describe the family caregiver as “co- patient” if he did not suffer from cancer disease; some authors see it as a “hidden patient” whose suffering is specific. But he is also a “co-therapist” of the patient. For Nijboer he can even be considered “primary caregiver”, it becomes a relay that allows caregivers to improve patients follow.

The limitations of our study is that the participants in the study might not represent the general population, as some patients immediately choose care in private centers, mainly those with a high socio-economic level. And maybe the impact in family caregivers would be different.

## Conclusion

In spite of the fact that there have been great impact on elderly cancer patient’s caregivers, the benefit of caregiving was observed in 80% in our study. Informal caregivers’ burden should be recognized by the society. Assistance and information from healthcare professionals remains the key to improve the ability of caregivers to cope with caring for elderly patients affected with cancer. More studies have to be conducted, especially in developing countries where the lack of resources increases the impact on family caregivers.

## Additional files

Additional file 1:
**Table S1.** Characteristics of the population. Additional file 2:
**Table S2.** Statistical analysis by non-parametric test to identify factors influencing the psychological impact on relatives of cancer patients.

## References

[1] Hudson P, Aranda S, McMurray N (2002). Intervention development for enhanced lay palliative caregiver support—the use of focus groups. Eur J Cancer Care.

[2] Schulz R, Gallagher-Thompson D, Haley WE, Czaja S, Schulz R (2000). Understanding the interventions process: a theoretical/conceptual framework for intervention approaches to caregiving. Handbook on dementia caregiving: evidence-based interventions for family caregivers.

[3] Moore K, Fortner B, Okon T (2003). The impact of medical visits on patients with cancer. Oncol Nurs Forum.

[4] Haley WE, Allen R, Reynolds S, Chen H, Burton A, Gallagher-Thompson D (2002). Family issues in end-of-life decision making and end- of- life care. Am Behav Sci.

[5] American Psychiatric Association (2003) In: DSM-IV-TR. Manuel diagnostique et statistique des troubles mentaux. Masson, Paris, p 1065

[6] Dilworth-Anderson P, Williams IC, Gibson BE (2002). Issues of race, ethnicity, and culture in caregiving research: a twenty-year review (1980–2000). Gerontologist.

[7] Haley W (2003). The costs of family caregiving: implications for geriatric oncology. Crit Rev Oncol Hematol.

[8] Balducci L, Extermann M (2000). Management of cancer in the older person: a practical approach. Oncologist.

[9] McMillan SC, Moody LE (2003). Hospice patient and caregiver congruence in reporting patients’ symptom intensity. Cancer Nurs.

[10] Braun M, Mikulincer M, Rydall A, Walsh A, Rodin G (2007). Hidden morbidity in cancer: spouse caregivers. J Clin Oncol.

[11] Janda M, Steginga S, Langbecker D, Dunn J, Walker D, Eakin E (2007). Quality of life among patients with a brain tumor and their carers. J Psychosom Res.

[12] McCorkle R, Siefert ML, Dowd MF, Robinson JP, Pickett M (2007). Effects of advanced practice nursing on patient and spouse depressive symptoms, sexual function, and marital interaction after radical prostatectomy. Urol Nurs.

[13] Matthews BA (2003). Role and gender differences in cancer-related distress: A comparison of survivor and caregiver self-reports. Oncol Nurs Forum.

[14] Mellon S, Northouse LL, Weiss LK (2006). A population-based study of the quality of life of cancer survivors and their family caregivers. Cancer Nurs.

[15] Foxall MJ, Gaston-Johansson F (1996). Burden and health outcomes of family caregivers of hospitalized bone marrow transplant patients. J Adv Nurs.

[16] Wallhagen MI (1992). Caregiving demands: their difficulty and effects on the well-being of elderly caregivers. Sch Inq Nurs Pract.

[17] Given CW, Given B, Stommel M, Collins C, King S, Franklin S (1992). The caregiver reactions assessment (CRA) for caregivers to persons with chronic physical and mental impairments. Res Nurs Health.

[18] Stetz KM (1987). Caregiving demands during advanced cancer: the spouse’s needs. Cancer Nurs.

[19] Stetz KM (1989). The relationship among background characteristics, purpose in life and caregiving demands on perceived health of spouse caregivers. Sch Inq Nurs Pract.

[20] Haley WE, LaMonde LA, Han B, Narramore S, Schonwetter R (2001). Family caregiving in hospice: effects on psychological and health functioning among spousal caregivers of hospice patients with lung cancer or dementia. Hosp J.

[21] Carey PJ, Oberst MT, McCubbin MA, Hughes SH (1991). Appraisal and caregiving burden in family members caring for patients receiving chemotherapy. Oncol Nurs Forum.

[22] Wallsten SS (2000). Effects of caregiving, gender, and race on the health, mutuality, and social supports of older couples. J Aging Health.

[23] Sales E, Schulz R, Biegal D (1992). Predictors of strain in families of cancer patients: a review of the literature. J Psychosoc Oncol.

[24] Mor V, Allen S, Malin M (1994). The psychosocial impact of cancer on older versus younger patients and their families. Cancer.

[25] Williamson GM, Shaffer DR, Schulz R (1998). Activity restriction and prior relationship history as contributors to mental health outcomes among middle-aged and older spousal caregivers. Health Psychol.

[26] Haley WE, Bailey S, Vellas B, Fitten JL (1999). Research on family caregiving in Alzheimer’s disease: implications for practice and policy. Research and practice in Alzheimer’s disease.

[27] Kiecolt-Glaser JK, Dura JR, Speicher CE, Trask OJ, Glaser R (1991). Spousal caregivers of dementia victims: longitudinal changes in immunity and health. Psychosom Med.

[28] Kiecolt-Glaser JK, Marucha PT, Malarkey WB, Mercado AM, Glaser R (1995). Slowing of wound healing by psychological stress. Lancet.

[29] Kiecolt-Glaser JK, Glaser R, Gravenstein S, Malarkey WB, Sheridan J (1996). Chronic stress alters the immune response to influenza virus vaccine in older adults. Proc Natl Acad Sci USA.

[30] King AC, Oka RK, Young DR (1994). Ambulatory blood pressure and heart rate responses to the stress of work and caregiving in older women. J Gerontol.

[31] Vitaliano PP, Russo J, Niaura R (1995). Plasma lipids and their relationships with psychosocial factors in older adults. J Gerontol B Psychol Sci Soc Sci.

[32] Schulz R, Beach SR (1999). Caregiving as a risk factor for mortality: the Caregiver Health Effects Study. JAMA.

[33] Hayman JA, Langa KM, Kabeto MU, Katz SJ, DeMonner SM, Chernew ME (2001). Estimating the cost of informal caregiving for elderly patients with cancer. J Clin Oncol.

[34] Houts PS, Lipton A, Harvey HA, Martin B, Simmonds MA, Dixon RH (1984). Nonmedical costs to patients and their families associated with outpatient chemotherapy. Cancer.

[35] Moore KA (1999). Breast cancer patients’ out-ofpocket expenses. Cancer Nurs.

[36] Varricchio C (1994). Human and indirect costs of home care. Nurs Outlook.

[37] Koop PM, Strang VR (2003). The bereavement experience following home-based family caregiving for persons with advanced cancer. Clin Nurs Res.

[38] Nijboer C, Triemstra M, Tempelaar R, Sanderman R, van den Bos GA (1999). Determinants of caregiving experiences and mental health of partners of cancer patients. Cancer.

