# Supplementary material for: The burden among family caregivers of elderly cancer patients: prospective study in a Moroccan population
Source: BMC Res Notes. 2015 Aug 13;8:347. doi: 10.1186/s13104-015-1307-5 (PMC4534123; doi:10.1186/s13104-015-1307-5)
Supplement: Additional file 1: — Table S1. Characteristics of the population. [file 13104_2015_1307_MOESM1_ESM.docx]

| **Characteristics** | **N** | % |  |  |
| --- | --- | --- | --- | --- |
| Mean Age (years +/- SD) | 44.74 ± 13.4 |  |  |  |
| Sex : |  |  |  |  |
| • man | 61 | 40.7 |  |  |
| • Women | 89 | 59.3 |  |  |
| Origin : |  |  |  |  |
| • urban | 100 | 66.7 |  |  |
| • Rural | 50 | 33.3 |  |  |
| Marital status | 94 | 62.7 |  |  |
| Number of children ( number +/- SD) | 3.33+/-2.34 |  |  |  |
| Normal physical health | 142 | 94.7 |  |  |
| Normal mental health | 150 | 100 |  |  |
| Economic situation | 93 | 62 |  |  |
| Employed |  |  |  |  |
| • Full time | 70 | 46.7 |  |  |
| • Half time | 16 | 10.7 |  |  |
| • Retired | 9 | 6 |  |  |
| Income: |  |  |  |  |
| • None | 55 | 36.7 |  |  |
| • < 200 euros | 39 | 26 |  |  |
| • 200 and 500 euros | 34 | 22.7 |  |  |
| • > 500 euros | 22 | 14.7 |  |  |
| Education |  |  |  |  |
| • Illiterate | 56 | 37.3 |  |  |
| • Completed | 94 | 62.7 |  |  |
| Distance |  |  |  |  |
| • In the same house | 80 | 53.3 |  |  |
| • More than an hour away | 43 | 28.7 |  |  |
| • Neighbors | 27 | 18% |  |  |

SD: standard deviation
