# Supplementary material for: The burden among family caregivers of elderly cancer patients: prospective study in a Moroccan population
Source: BMC Res Notes. 2015 Aug 13;8:347. doi: 10.1186/s13104-015-1307-5 (PMC4534123; doi:10.1186/s13104-015-1307-5)
Supplement: Additional file 2: — Table S2. Statistical analysis by non-parametric test to identify factors influencing the psychological impact on relatives of cancer patients. [file 13104_2015_1307_MOESM2_ESM.doc]

**Table S2: Statistical analysis by non-parametric test to identify factors influencing the psychological impact on relatives of cancer patients**

|  | | **depression** | | **anxiety** | | **Fear of**  **contagion** | | **Fear of**  **inheriting cancer** | |
| --- | --- | --- | --- | --- | --- | --- | --- | --- | --- |
|  |  | **%** | **p** | **%** | **p** | **%** | **p** | **%** | **p** |
| **Sex** | **Male**(n=61) | 51.8 | <0.001 | 60.2 | <0.001 | 72.4 | 0.17 | 61.4 | 0.002 |
|  | **Female** (n=89) | 91.7 |  | 85.9 |  | 77.6 |  | 83.6 |  |
| **origin** | **Urban** (n=100) | 82.5 | 0.001 | 81.2 | 0.001 | 69.7 | <0.001 | 77.7 | 0.068 |
|  | **Rural** (n=50) | 61.5 |  | 64 |  | 87 |  | 68 |  |
| **School level** | **Education completed**  (n=94) | 76.3 | 0.704 | 75.8 | 0.85 | 69.1 | <0.001 | 74.9 | 0.82 |
|  | **Illiterate** (n=56) | 74 |  | 74.9 |  | 86.2 |  | 73.8 |  |
